# AI powered patient records analysis for injury surveillance in children and adolescents – a feasibility study from Switzerland

**DOI:** 10.3389/ijph.2026.1609472

**Published:** 2026-08-19

**Authors:** Sonja Feer, Felix Matthias Saaro, Annina Zysset, Mirjam Bächli, Steffen Nieman, Delphine Meier, Michelle Seiler, Jasmina Bogojeska, Julia Dratva

**Affiliations:** 1 Institute of Public Health, Zurich University of Applied Sciences, Winterthur, Switzerland; 2 Centre for Artificial Intelligence, Zurich University of Applied Sciences, Winterthur, Switzerland; 3 Swiss Council for Accident Prevention BFU, Bern, Switzerland; 4 Pediatric Emergency Department and Children’s Research Centre, University Children’s Hospital Zurich, Zurich, Switzerland; 5 Medical Faculty, University of Basel, Basel, Switzerland

**Keywords:** artificial intelligence, children and adolescents, injury, prevention, surveillance

## Abstract

**Objective:**

This study investigates the feasibility of using an automated approach to extract injury-related information from narrative text in a paediatric emergency department to improve data basis on injuries among children and adolescents in Switzerland.

**Methods:**

The dataset comprises paediatric injury cases treated between 2018 and 2022 at the University Children’s Hospital Zürich emergency department (N = 30,876). Model development involved (1) adapting EU-IDB, a domain-specific hierarchical *coding-system*; (2) *manual data annotation*; and (3) *fine tuning* and (4) *evaluation* of a transformer-based model for multi-label text span classification.

**Results:**

Interrater reliability improved from moderate (κ = 0.45) to substantial (κ = 0.73) following adaptation of the coding system. Variable level classification performance was encouraging across three training variants (macro F1: ALL = 0.57, IND = 0.64, VAR = 0.63). Performance declined at lower levels, particularly at level 3 (macro F1: ALL = 0.08, IND = 0.23). Automated prevalence estimates correlated strongly with manual annotations (ρ = 0.948).

**Conclusion:**

Automated text span classification of injury-relevant information from electronic patient records shows promising first results to provide valuable information for injury surveillance and prevention, missing in Switzerland. However, more annotated data and detailed validation are needed to draw robust conclusions.

## Introduction

Injuries in the age group of children and adolescents are a major public health concern. Unintentional injuries cause nearly 90% of total injury cases and are a primary reason for death and disability among the age group of children and adolescents [1]. Many injured children suffer from lifelong consequences such as disabilities or scarring [1]. Accidents also demand substantial healthcare resources, as injured children often require acute medical care [2]. Therefore, prevention of injuries must be a major public health initiative to enhance health and wellbeing of children and reduce the healthcare burden.

In Switzerland, data on child injuries is both incomplete and fragmented [3]. Existing data from various national reporting systems – such as road traffic accident reports, mortality statistics, or poison control services – provide only limited insight into the actual extent and causes of accidents and injuries among children and adolescents. Representative data on non-fatal injuries in the home and leisure sector and their causes are insufficiently available or not accessible in Switzerland [4]. Moreover, existing data sources also vary considerably in the level of detail provided. However, detailed information on injuries, in particular injury circumstances, e.g., place of occurrence, activity when injured or mechanism of injury, are important information for evidence-based injury prevention [5]. In addition to repeated national surveys, systematically collected clinical data on injuries would provide relevant and timely information.

In many countries, clinical data is used for injury surveillance in children and adolescents. A well-documented example is the Styrian Injury Surveillance System (StLSS) in Austria, which has been collecting detailed data on injuries among child and adolescents [6, 7]. Clinical data is also utilized in the European Injury Database (EU-IDB), to record injuries among children and adolescents in a standardized format across participating countries, based on emergency department (ED) visits [8]. Similarly, both the Canadian Hospitals Injury Reporting and Prevention Program (CHIRPP) and the National Electronic Injury Surveillance (NEISS) in the USA collect injury data on children and adolescents in EDs [9, 10]. These injury surveillance systems based on clinical data from hospitals have traditionally depended on manual data entry and interpretation [11].

Recent advancements in Machine Learning (ML), namely, Large Language Models (LLMs), offer a promising approach to enhance injury insights by enabling the automatic classification of injury-relevant information from already existing narrative (unstructured) clinical text data. Vallmuur et al. concluded that applying ML techniques, to narrative text data can improve the completeness and timeliness of injury surveillance and thereby support more effective injury prevention policies and practices [12]. Recently, Azzolina et al. (2023) trained a model based on a random sample of manually annotated narrative diagnosis texts and concluded that ML methods are promising for improving injury surveillance by automatically classifying paediatric ED diagnoses [13]. Moreover, automated approaches have the potential to identify risk factors as basis for tailored health prevention strategies [14]. International efforts demonstrate that these automated approaches are highly efficient [12, 13, 15–17].

Automated approaches have been successfully applied for screening or classification of a single injury-related information from narrative clinical text data for surveillance tasks. To our knowledge, so far only one study by Choi et al. has managed to successfully classify a comprehensive set of injury-related information from ED narrative clinical text data [17]. They fine-tuned an LLM (Llama-2 12B parameters) on a multi-label task to classify five variables with 24 sub-labels from a large injury dataset, covering ED cases from two adult cohorts. However, an attempt at multi-label text-span classification to obtain injury-relevant information from unstructured ED narrative text data using automated approaches has not been published to date.

This study investigates the feasibility and necessary validation steps of this approach, applying an automated approach to classify a comprehensive set of injury-related information, using narrative (unstructured) text data recorded by medical staff in a paediatric ED. It is assumed that potentially valuable injury-related information–such as place of occurrence, mechanism of injury, and objects involved - is contained within these narrative text data. Combined with already structured data from electronic patient records (e.g., age and sex), this approach aims to not only improve the surveillance data on injuries in children and adolescents in Switzerland but also to support evidence-based injury prevention measures for children and adolescents.

## Methods

### Study population and data

Electronic health record data were accessed from ED visits from the University Children’s Hospital Zurich (UCHZ). All injury cases of children and adolescents aged 0 to 18, who were treated for injury between 1.1.2018 and 31.12.2022 at the UCHZ ED and for whom a general consent was obtained were included in the study. Data was anonymized by the UCHZ prior to the data transfer, ensuring that no personally identifiable data or identification codes, allowing data to be traced back to a patient, were included in the dataset. Ethics application was submitted to the Ethics Committee of the canton of Zurich (BASEC-Nr.: 2023-01947) and the project was approved.

Data include: Reason for treatment (injury vs. illness) recorded as a structured variable upon admission in the ED, further variables such as age (in years), sex, and month/year of arrival, as well as unstructured narrative data, such as a diagnosis and medical history text. To ensure data accuracy and consistency, a data cleaning process was conducted prior to analysis. The data cleaning involved exclusion of cases with repeated visits for the same injury (n = 4,270), missing key variables such as age, sex and month/year of treatment (n = 4), fell outside the observation period (n = 7), outside age range (n = 2) or duplicates (n = 6). The final dataset comprised 30,876 injury cases of children and adolescents aged 0–18 years, with a mean age of 6.9 years (SD 4.5).

**FIGURE 1:**
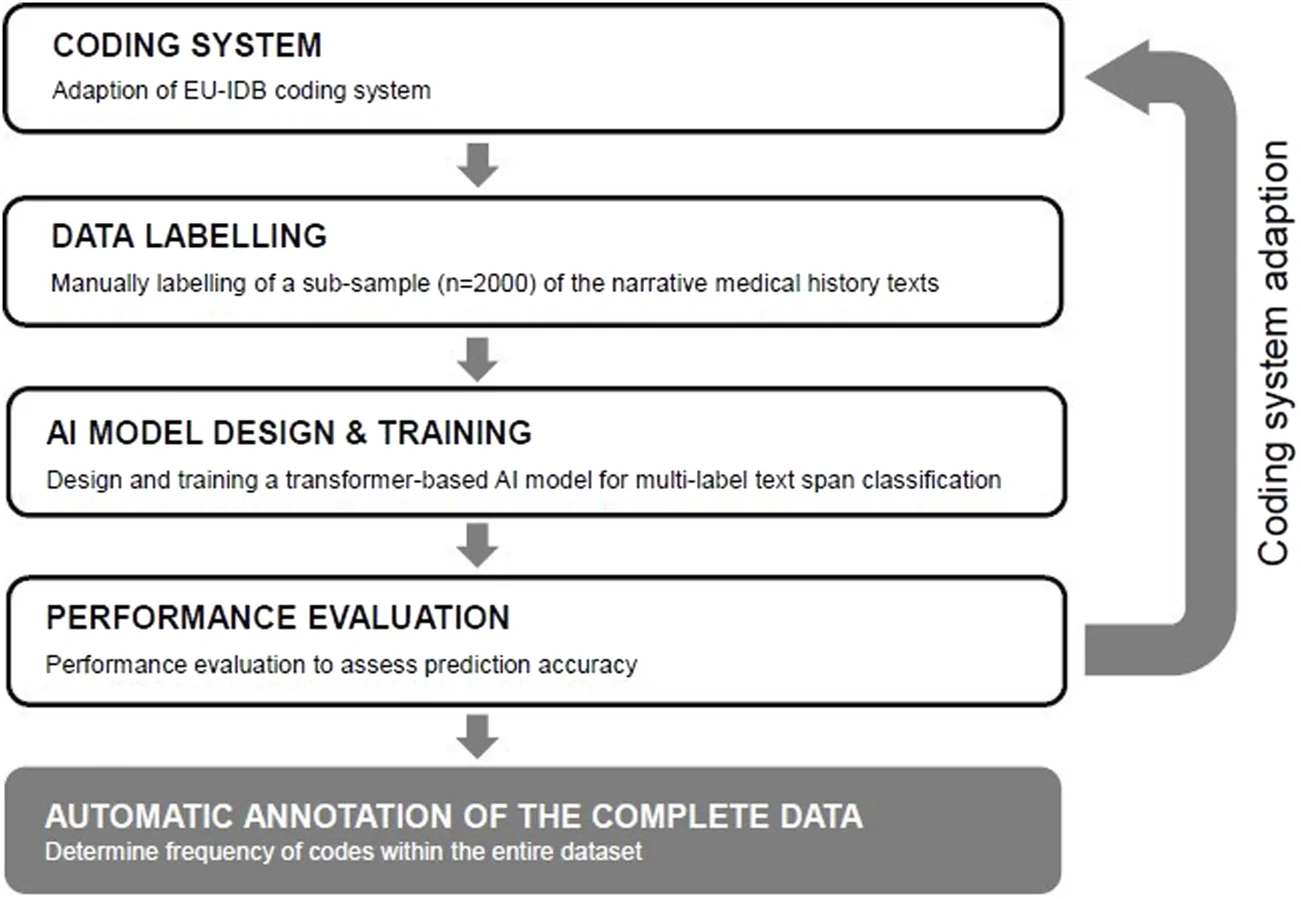
Steps of model development and application. Switzerland, 2018–2022.

This study followed a structured iterative process for the model development that involved various steps (see Figure 1) and adopted a cyclical workflow, enabling stepwise refinement through continuous learning and evaluation. The iterative process facilitated dynamic adaption to new insights based on evaluation and testing, addressed challenges as they emerged, and progressively enhanced study outcome.

### Coding system

The internationally established injury coding system EU-IDB was chosen to label injury-relevant information [18]. It has a hierarchical structure, in which each variable can be further specified in up to two or three levels of labels (e.g., variable *place of occurrence* - Level 1 *private living area* - Level 2 *kitchen*) [18]. It covers all age groups including relevant injury labels for children and adolescents. After the initial manual annotation phase and preliminary classification results, the coding system was substantially reduced and adapted to better reflect injury patterns in children and adolescents, such as reducing the number of labels and adding child-specific labels, reducing the labels by a third from a total of 1187 to 574. The final coding system comprised the variables *place of occurrence*, *mechanism of injury*, *activity when injured*, *products involved*, *type of sport* and *mode of transportation,* as well as *height,* added by the authors to obtain additional information about the accident circumstances.

### Manual annotation

To create a dataset for the model training and evaluation, a randomly chosen subset of the narrative medical history texts was manually annotated. The variables and their corresponding labels were assigned to identified text spans which provided relevant information for a specific variable of interest (see Table 1). For the annotation process, the tool Prodigy (https://prodi.gy/) was used. Manual annotation was done in two phases by a total of five individuals (annotators): three members of the project team, and two scientific assistants. All annotators received training that included an introduction to the coding tree and coding rules, followed by practical exercises in which randomly selected cases were annotated and discussed in the group.

**TABLE 1:** Example manual annotation narrative medical history text. Switzerland, 2018–2022.

Narrative medical history text				
Child was playing with her sibling on a loft bed in the bedroom and suddenly fell down (approx. 110 cm). She suffered RQW on the left eyelid. Cried straight away, was not unconscious, no vomiting so far, played at home				

Annotations (identification of text spans and assignment of labels)				
Text span	Label - variable	Lable - level 1	Lable - level 2	Lable - level 3
Playing	Activity when injured	Leisure activity	Playing	​
Loft bed	Product	Furniture and furnishings	Bed	​
Bedroom	Place of occurrence	Home	Living room, bedroom, children’s room	N/A
Fell down	Mechanism of injury	Falling, stumbling, jumping, pushed	N/A	​
110 cm	Height	Falling, stumbling, jumping, pushed (>0.5 m and <1.5 m)	​	​

The degree of agreement between the annotators was analysed twice during the feasibility study. For each test, a sample of 50 cases was randomly selected from the entire dataset and independently manually annotated by each annotator. Cohens κ was used to assess interrater reliability, where agreement is defined as both annotators selecting the same variable and producing overlapping text spans. In the first annotation phase, two annotators manually annotated n = 1,000 cases using the original IDB coding tree. The first interrater test involved three annotators, of which one was considered as the reference annotator because of his established experience in applying the IDB coding tree. The first interrater reliability showed a moderate agreement (κ = 0.45). Given the changes to the coding system, two new annotators were trained and involved in the second manual annotation phase, which comprised the manual annotation of an additional n = 1,000 cases and re-annotation of the initial n = 1,000 cases to reflect updated coding rules. This resulted in a final dataset (n = 2,000) single-annotated according to the same coding system. The new annotators performed a second interrater test using the adapted coding tree, resulting in substantial agreement between annotators (κ = 0.73).

To evaluate the performance of the trained models the manually annotated dataset was randomly divided into a training set (n = 1,800) and a test set (n = 200).

### Model design and training for classification

The base model, an instruction fine-tuned version of the Mistral 7B v0.3 model (released May 22, 2024), was chosen because of its multilingual ability, openly available weights, and low hardware requirements for the fine-tuning. It is a transformer model with approximately seven billion parameters [19]. Supervised fine-tuning was performed using Parameter-Efficient Fine-Tuning (PEFT) via the Low-Rank Adaptation (LoRA) method [20]. Details regarding the hyperparameters can be found on the project page (https://github.com/AIPRA-IS/feasibility). Fine-tuning was formulated as a multi-label text-span classification task. During training, the model is provided with ED texts together with a semi-structured prompt that highlights the target variable and instructs the model to extract the corresponding spans. The model then produces a structured output that contains both the extracted text span and its associated hierarchical labels (levels 1–3) in the same structure as the annotated dataset in Table 1. This output format ensures that the model does not only locate the relevant span but also assigns the appropriate variable. Training was conducted on a single NVIDIA Tesla V100 GPU. Three variations of the fine-tuning setup were implemented to compare different prediction targets:

- ALL One model to find text spans and assign the correct variable as well as the labels from the hierarchical coding system.
- VAR One model to find text spans and only assign the correct variable.
- IND A separate model for each variable (variable-specific model) to find the text spans and assign the labels from the hierarchical coding system for the target variable.

### Performance evaluation

Model performance was assessed in two steps. First, for each ground-truth text span, we checked whether the model produces a text span sharing at least one word with it. Second, for each such overlapping span, we verified whether the predicted Variable and Level 1–3 labels are correct. This enables the generation of a confusion matrix for each label at each level, from which an F1 score is computed that is especially suitable for unbalanced class distributions.

To obtain uncertainty estimates for the performance scores without requiring costly model training, we applied non-parametric bootstrapping by resampling the test documents with replacement over 1,000 iterations, computing the F1 score in each iteration to derive 95% confidence intervals.

### Automatic annotation of the complete data

To determine the frequency of each code within the entire dataset, the IND model, chosen because it performed best across the hierarchical levels, was applied to estimate the prevalence of all hierarchical labels for each variable separately. However, applying a classifier directly to estimate prevalence via naïve counting is known to produce biased estimates [21], as asymmetric misclassification rates cause systematic over- or underestimation of class frequencies. To correct for this, we calibrated the raw prediction counts using the confusion matrix derived from the annotated subset and the Bayesian Classify and Count (BCC) method proposed by von Däniken et al. (2024) [22], which adjust prevalence estimates to account for the classifier’s error structure. To assess plausibility, we compared the prevalence estimates obtained from the automatic annotations with those from the manually annotated subset.

## Results

### Manually annotated data

Our subset of 2,000 manually annotated narrative medical history texts contained a total of 5,326 annotations, each containing a text span with the corresponding variable and labels from the coding system. Table 2 shows the utilization of the labels per levels (coding hierarchy). Level 1 and 2 show a high utilization with 92.6% respectively 71.3%, although usage is lower for the variables *product* and *sport* for level 2. In contrast, level 3 shows lower utilization, with 53.9% of the available labels being used.

**TABLE 2:** Number of available (Av), utilized (Util), and annotated (Annot) labels in the annotated dataset (n = 2,000 narrative medical history texts) for each level grouped by the variable including the utilization across each level. Switzerland, 2018–2022.

Variable	Level 1			Level 2			Level 3		
	Av	Util	Annot	Av	Util	Annot	Av	Util	Annot
Place of occurrence	9	9	750	36	32	649	17	14	96
Mechanism of injury	10	9	1858	33	27	1213	24	10	95
Activity when injured	8	8	955	28	25	735	0	0	0
Product	17	17	1090	76	58	918	197	105	734
Sport	17	13	338	69	31	290	0	0	0
Mode of transportation	4	4	169	16	11	169	3	1	4
Height	3	3	161	0	0	0	0	0	0
Total #	68	63	5321	258	184	3974	241	130	929
Utilization %	92.6			71.3			53		

Table 2 also shows the annotation counts per level grouped by variables. *Mechanism of injury* is the most often used variable with a total of 1,859 annotations. Of these n = 1,858 have an annotation on level 1, n = 1,213 on level 2 and n = 95 on level 3.

The number of annotations, and the number of labels used indicate that annotations of variables and level 1 are frequent. On level 2 the number of annotations is still high (n = 3,790) but spread across a lot of labels (n = 184). Level 3 has fewer annotations (n = 929) but still comprising many labels (n = 130), resulting in a lower number of annotations per code.

### Performance evaluation

**FIGURE 2:**
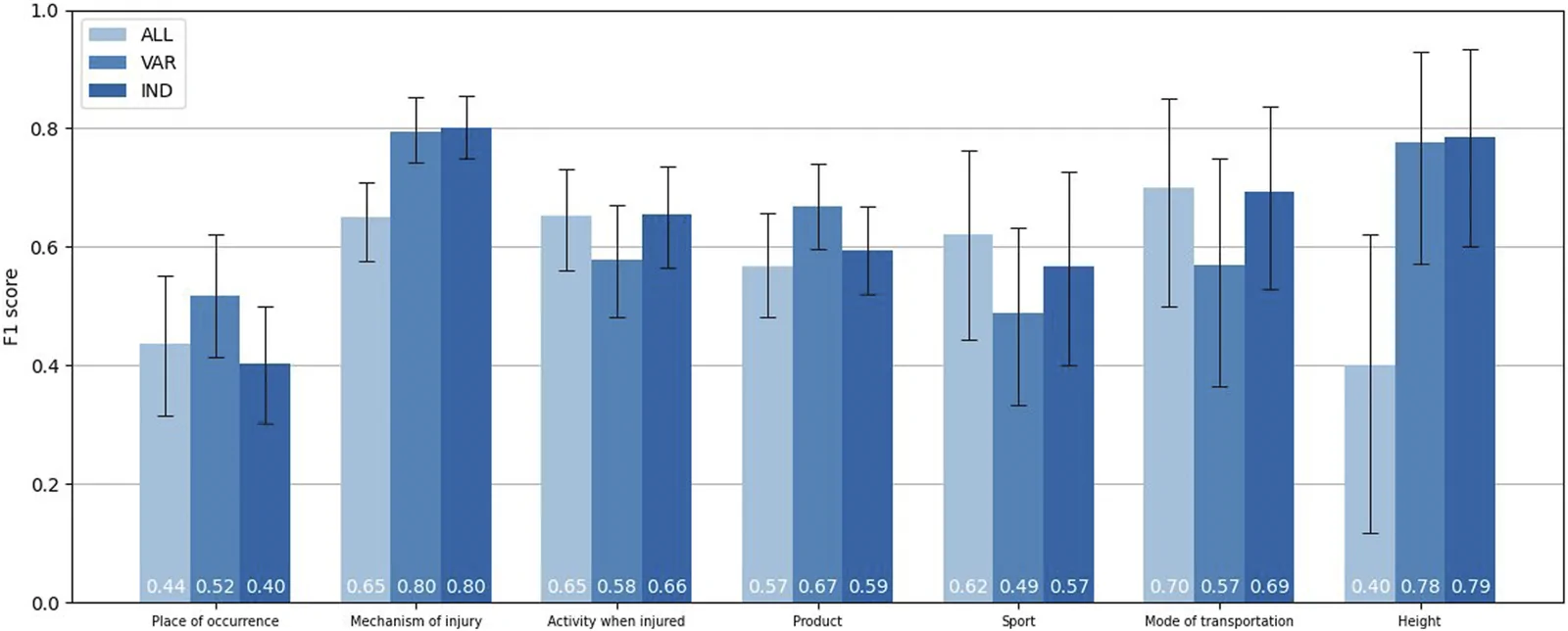
F1 score and 95% confident interval of the variable classification for the variants ALL (one model trained on all variables and levels), VAR (one model trained on only the variables without the hierarchical labels), and IND (individually trained model for each variable). Switzerland, 2018–2022.

Figure 2 shows the classification performance across the three training variations (ALL, VAR and IND) including the 95% confidence intervals. Prediction performance on *mechanism of injury,* the variable with the most annotations*,* was high across all three variations (ALL = 0.65, VAR = 0.795, IND, 0.80) with the smallest confidence intervals. The models VAR and IND showed a good performance on *height* (VAR = 0.77, IND = 0.78) while ALL exhibited the lowest performance (ALL = 0.4) compared to all other variables. The confidence intervals on *height* were also the largest and it is the class with the lowest number of annotations. Fine-tuning a model to predict only the variable (VAR) yielded better average performance across most variables (Macro F1 = 0.63), however, it seemed to work worse for the variables *sport*, *mode of transportation*, and *activity when injured*. The best performance on average was achieved by separately fine-tuning individual variable-specific models (IND), with an average Macro F1 score of 0.64.

Table 3 shows the macro F1 scores for variable-level and hierarchical code-level prediction across the three model configurations (ALL, VAR, IND). At the variable level, the IND and VAR models performed comparably, and both outperformed the ALL model (mean F1: IND = 0.64, VAR = 0.63, ALL = 0.57). Gains were most pronounced for specific variables. The IND model substantially improved prediction of *height* (F1 = 0.78 vs. 0.39 for ALL) and *mechanism of injury* (F1 = 0.80 vs. 0.65), while the VAR model showed the strongest performance for product (F1 = 0.67) and achieved F1 = 0.78 for *height*. Across all models, prediction performance declined with each lower level of the label hierarchy. At level 1, the IND model again outperformed ALL (mean F1: 0.40 vs. 0.32). At level 2, performance was considerably lower across all variables (IND mean F = 0.26, All mean F1 = 0.23), with *mechanism of injury* showing the greatest benefit from the IND configuration (F1 = 0.42 vs. 0.23). Level 3 prediction was largely poor or not applicable. *Mechanism of injury* achieved F1 = 0.00 under the ALL model and only 0.28 under IND, while *mode of transportation* returned F1 = 0.00 under both configurations. Across all three model types, *mechanism of injury* and *mode of transportation* at the variable level, and *height* at level 1, showed the strongest absolute performance, whereas level 3 prediction across all variables remained the most challenging aspect of the classification task.

**TABLE 3:** F1 scores and 95% confidents intervals for the variants ALL (one model trained on all variables and levels) and IND (individually trained model for each variable) across the Variable and Label 1 to 3 aggregated by variable. Switzerland, 2018–2022.

​	Variable			Level 1		Level 2		Level 3	
Variable	ALL	IND	VAR	ALL	IND	ALL	IND	ALL	IND
Place of occurrence	0.43	0.40	0.52	0.28	0.25	0.26	0.19	0.17	0.41
	0.31–0.55	0.30–0.50	0.42–0.62	0.16–0.43	0.16–0.36	0.15–0.36	0.10–0.28	0.00–0.50	0.00–0.83
Mechanism of injury	0.65	0.80	0.80	0.44	0.57	0.23	0.42	0.00	0.28
	0.58–0.71	0.75–0.86	0.74–0.85	0.31–0.60	0.42–0.79	0.18–0.29	0.32–0.52	0.00–0.00	0.00–0.59
Activity when injured	0.65	0.66	0.58	0.3	0.33	0.23	0.26	​	​
	0.56–0.73	0.57–0.74	0.48–0.67	0.22–0.41	0.23–0.45	0.15–0.33	0.17–0.37	​	​
Product	0.57	0.60	0.67	0.33	0.36	0.28	0.27	0.14	0.23
	0.48–0.66	0.52–0.67	0.60–0.74	0.25–0.42	0.25–0.46	0.20–0.37	0.19–0.35	0.08–0.20	0.15–0.31
Sport	0.62	0.57	0.49	0.22	0.23	0.13	0.13	​	​
	0.44–0.76	0.40–0.73	0.33–0.63	0.10–0.36	0.11–0.39	0.09–0.21	0.07–0.21	​	​
Mode of transportation	0.69	0.69	0.57	0.43	0.36	0.22	0.28	0.00	0.00
	0.50–0.85	0.53–0.84	0.36–0.75	0.29–0.83	0.25–0.69	0.10–0.40	0.17–0.45	0.00–0.00	0.00–0.00
Height	0.39	0.78	0.78	0.24	0.68	​	​	​	​
	0.12–0.62	0.60–0.93	0.57–0.93	0.00–0.50	0.43–0.88	​	​	​	​
Average	0.57	0.64	0.63	0.32	0.40	0.23	0.26	0.08	0.23

### Automatic annotation of the complete data

Table 4 shows the prevalence (including the estimated standard deviation) of the most relevant labels (prevalence at least 1%) of level 1 and 2 labels grouped by their variable within the annotated subset (n = 2,000) and compares it to the entire dataset (n = 30,876). The prevalence from the manual annotations is compared to the prevalence derived from the predictions obtained by the IND models on the full dataset. This comparison yields a Spearman correlation coefficient of ρ = 0.948 across the labels listed in Table 3.

**TABLE 4:** Prevalence estimates of the labels within the annotated dataset (n = 2,000) and the predictions of the entire dataset, including their difference, from the ED of the University Children’s Hospital Zurich (UCZH) (n = 30,876). Switzerland, 2018–2022.

Variable	Annotated Dataset		Entire Dataset		​
​	Prevalence	Std. Dev.	Prevalence	Std. Dev.	Diff
Mechanism of injury					
1 – Blunt force	30%	±1.0%	26%	±0.2%	4%
1.2 – Contact with object/material/element	16%	±0.8%	18%	±0.2%	−2%
1.3 – Contact with person	6%	±0.5%	2%	±0.1%	4%
2 – Falling, stumbling, jumping, pushed	49%	±1.1%	46%	±0.3%	3%
2.1 – Tripping, stumbling	6%	±0.5%	4%	±0.1%	2%
2.2 – Slipping, sliding	5%	±0.5%	3%	±0.1%	2%
2.6 – Fall from/with sports/transport equipment	4%	±0.4%	1%	±0.1%	3%
4 – Thermal mechanism	3%	±0.4%	2%	±0.1%	1%
4.1 – Heat	3%	±0.4%	2%	±0.1%	1%
7 – Physical overexerting	8%	±0.6%	6%	±0.1%	2%
7.1 – Acute overexertion	8%	±0.6%	6%	±0.1%	2%
Product					
5 – Furniture and furnishings	9%	±0.6%	8%	±0.2%	1%
5.1 – Bed (incl. parts/components)	2%	±0.3%	1%	±0.1%	1%
5.2 – Chair, bench, armchair, sofa	4%	±0.4%	2%	±0.1%	2%
6 – Baby equipment or children’s product	10%	±0.7%	16%	±0.2%	−6%
6.1 – Baby or children’s item	2%	±0.3%	2%	±0.1%	0%
6.2 – Toys	1%	±0.2%	1%	±0.0%	0%
6.3 – Play equipment	7%	±0.6%	8%	±0.2%	−1%
10 – Sports equipment	7%	±0.6%	5%	±0.1%	2%
14 – Building structure or component	9%	±0.6%	5%	±0.1%	4%
16 – Material (n.e.c.)	3%	±0.4%	5%	±0.1%	−2%
Activity when injured					
3 – Education (incl. school sport, university sport)	6%	±0.5%	3%	±0.1%	3%
4 – Sport and physical activity	18%	±0.9%	12%	±0.2%	6%
5 – Leisure activity	16%	±0.8%	15%	±0.2%	1%
5.2 – Playing	14%	±0.8%	13%	±0.2%	1%
8 – Being on the move, journeys/transports	3%	±0.4%	5%	±0.1%	−2%
Place of occurrence					
1 – Home	13%	±0.8%	3%	±0.1%	10%
1.2 – Living room, bedroom, children’s room	6%	±0.5%	1%	±0.1%	5%
4 – Day care, school, educational area	12%	±0.7%	6%	±0.1%	6%
4.1 – School or educational establishment	5%	±0.5%	2%	±0.1%	3%
4.2 – Day care facility	3%	±0.4%	1%	±0.0%	2%
5 – Sports and athletics area	7%	±0.6%	1%	±0.1%	6%
6 – Transport area	2%	±0.3%	1%	±0.0%	1%
10 – Recreational area	2%	±0.3%	1%	±0.0%	1%
Sport					
1 – Team ball sport	8%	±0.6%	6%	±0.1%	2%
1.9 – Football (soccer)	6%	±0.5%	4%	±0.1%	2%
8 – Artistic gymnastics and sport gymnastics with equipment	1%	±0.2%	1%	±0.1%	0%
17 – Wheeled sport (non-motorised)	4%	±0.4%	1%	±0.0%	3%
Height					
1 – Falling, stumbling, jumping, pushed (<0.5 m)	2%	±0.3%	4%	±0.1%	−2%
2 – Falling, stumbling, jumping, pushed (≥0.5 m and <1.5 m)	4%	±0.4%	4%	±0.1%	0%
3 – Falling, stumbling, jumping, pushed (≥1.5 m)	2%	±0.3%	2%	±0.1%	0%
Mode of transportation					
1 – Transport means without motor drive	8%	±0.6%	4%	±0.1%	4%

For most labels, the difference between the prevalence estimations obtained from the annotated dataset and those obtained from the entire dataset is small. However, notable absolute discrepancies exist for certain labels, such as *product (baby equipment or children’s product: 6%), activity when injured (sport and physical activity: 6%), place of occurrence (home: 10%)*.

## Discussion

This study investigated the feasibility of utilizing an automated approach to extract multiple injury-related information from narrative (unstructured) medical history texts from a paediatric ED. Three fine-tuning model variants were evaluated: a single model predicting all variables and labels simultaneously (ALL), a model predicting only the variable label (VAR), and separate variable-specific models (IND). Variable-level extraction performance was encouraging across all three variants, demonstrating that the narrative texts contain sufficient injury-relevant information for automated extraction, consistent with our prior utility evaluation [3].

Applying the IND model to the full dataset produced prevalence estimates that showed a strong rank correlation with the manually annotated subset (ρ = 0.948), and are broadly consistent with national injury projections and international data, for instance, *falls* as the leading mechanism of injury in children and adolescents [23, 24]. Notable absolute discrepancies exist for certain labels (*product, activity when injured and place of occurrence).* While the precise sources cannot be determined with certainty, these discrepancies may reflect a combination of sampling bias in the annotated subset, systematic under- or over-representation of certain injury contexts in the narrative texts, and the model’s tendency to misclassify semantically similar labels, particularly in categories where training annotations were sparse. The consistency with established epidemiological patterns is encouraging as a plausibility check, but larger and more representative annotated datasets are needed before the model’s use for surveillance purposes.

The hierarchically structured coding system, with its large number of labels across multiple levels, presented a fundamental challenge for automated classification. Adapting the EU-IDB coding system to the paediatric context, was a key prerequisite for model development. Reducing the breath of labels not relevant to children and adolescents improved interrater reliability. However, performance degraded consistently at lower levels of the coding hierarchy across all model variants, with the sharpest drop observed at level 3 (see Figure 2). Reflecting on one side the structural limitation of the data providing limited level of detail present in the ED narrative texts for some variables (e.g., *injury circumstances*) and greater detail for others (e.g., *product*). Highlighting a fundamental tension between the granularity of information needed for evidence-based injury prevention and the level of detail routinely captured in clinical documentation [25]. On the other side, the small number of manual annotations (n = 2,000) illustrates the challenge of “data annotation bottleneck” as identified as a key obstacle in NLP-based automatic extraction approaches in other clinical text data more broadly [26]. Small training sets also increase the risk of overfitting, limiting the model’s ability to learn robust patterns from rare labels [27]. In addition, confidence intervals indicate substantial uncertainty for variables with few annotations. This is an expected consequence of the small test set (n = 200) and the uneven label distribution, limiting the strength of conclusions that can be drawn. The number of manual annotations in our feasibility study produced a dataset of sufficient quality for model training to establish important first insights. However, a larger annotated dataset with sufficient examples for lower levels is needed to draw robust conclusions about model performance across the full label space. Underscoring the trade-off between coding granularity, necessary for detailed injury surveillance, and the annotation effort required to achieve reliable model performance at lower levels.

Historically, injury surveillance has relied on manual data entry and interpretation. Applying automated extraction to ED electronic records has the potential to establish comprehensive injury surveillance systems that minimise manual effort while delivering high accuracy [15–17, 28, 29]. Such integration could transform how injury data are collected and used, enabling timely surveillance and more efficient allocation of healthcare resources, ultimately strengthening public health responses and improving patient health outcomes [30]. International efforts in countries such as Canada and Italy are also attempting to implement AI-based approaches for paediatric injury surveillance [11, 13]. However, unlike Switzerland, those countries already possess substantial historically annotated injury datasets to draw on.

To our knowledge, this is one of the first studies to classify multiple injury-related text spans from paediatric ED records using a hierarchical coding system, extending beyond the single-variable classification approaches that characterise most prior work [11, 13, 17]. Preserving text span context improves interpretability and opens opportunities for automated data enrichment. Future integration of explainable AI techniques for LLMs could further strengthen transparency and clinical trust in model outputs [31], addressing one of the key barriers to adopting AI in clinical and public health settings [30].

### Limitations

Several limitations of this feasibility study should be acknowledged:

First, the complexity of the EU-IDB coding tree posed a substantial challenge for automatic classification. Despite reducing the original coding system, the remaining hierarchical structure proved difficult for the models to navigate, particularly at lower levels where prediction performance declined markedly. Future work should explore whether further reduction of the coding tree, guided by annotation density and surveillance priorities, could improve classification performance without sacrificing the detail need for evidence-based injury prevention.

Second, the small number of manual annotations limited the size of the training and test datasets. To draw robust conclusions about the automated extraction, further evaluation using a larger test dataset with sufficient sample size for all analysed label categories is required. This highlights the necessity of increasing the manually annotated data to provide the basis for further development and enhancements of automated approaches for injury monitoring.

Third, the choice of model architecture and training setup involved trade-offs that may have affected performance. Only a single base model was used for the evaluation. Future work should compare different model sizes and output generation approaches such as reasoning strategies, unavailable at the time of the study. In addition, this baseline should be used to identify which narrative annotations would most improve classification performance. In this way, the manual annotation effort can be optimized so that only as much data is manually annotated as necessary to achieve a useful prediction performance.

Finally, model evaluation was limited in scope. Performance was assessed on a small test set, without cross-validation, which affects the robustness of the reported estimates for labels with a small number of samples in the test set. Further evaluation using human annotations of a blinded sample is needed to distinguish whether limitations stem from insufficient annotations, fine-tuning shortcomings, or inherent text ambiguity.

### Conclusion

Our feasibility study provides important first insights on the automatic identification and multi-label classification of text spans containing paediatric injury-relevant information in Switzerland. This approach has the potential to improve currently insufficient injury surveillance and evidence-based prevention in children and adolescents in Switzerland. Prerequisites for the successful application of automated systems include digital access to ED patient records and detailed narrative text input from medical staff.

The results demonstrate that reducing its complexity and adding child-specific labels to the EU-IDB coding tree, enhanced model performance. The remaining hierarchical structure continued to pose challenges. Future work should expand the annotated dataset, systematically evaluate alternative models, integrate most recent methodological advances, and explore continuous active learning strategies to optimise the annotation effort. Model evaluation using larger test sets, evaluation across multiple representative test sets, cross-validation when feasible, and human validation will be required before it can be considered as ready for implementation in child injury surveillance in Switzerland. Data privacy, ethical considerations and transparency are other challenges that must be carefully addressed to ensure a secure and responsible implementation of the approach in using clinical data. Considering these challenges and once the necessary advancements for implementation are achieved, applying automated methods for text span classification while preserving injury context information could enable timely surveillance of injury trends allowing rapid responses to emerging injury patterns and circumstances and thereby supporting evidence-based prevention strategies aimed at improving public health outcomes in children and adolescents.
